# The Influence of Stress and Anxiety on the Expectation, Perception and Memory of Dental Pain in Schoolchildren

**DOI:** 10.3390/dj6040060

**Published:** 2018-10-22

**Authors:** Gabriela de A. Lamarca, Mario V. Vettore, Angela M. Monteiro da Silva

**Affiliations:** 1 Centre of Studies, Policies and Information on Social Determinants of Health, National School of Public Health, Oswaldo Cruz, Foundation, Rio de Janeiro 21041-210, Brazil; 2 Academic Unit of Oral Health, Dentistry and Society, University of Sheffield, Sheffield S10 2TA, UK; m.vettore@sheffield.ac.uk; 3 Academia Brasileira de Ciências e Artes Orientais, Av Nossa Senhora de Copacabana, 928, 5º andar, Copacabana, Rio de Janeiro 22060-002, Brazil; angelamonteiro@gmail.com

**Keywords:** paediatric dental pain, stress, state and trait anxiety

## Abstract

The aim of this study was to investigate the association of stress and anxiety with the expectation, perception and memory of dental pain among schoolchildren. A follow-up study involving 46 children aged 9 to 12 years was conducted in a public school in the city of Petropolis (RJ), Brazil. Demographic characteristics, stress (children’s stress scale), and state and trait anxiety (state–trait anxiety inventory) were recorded before a dental procedure to restore the occlusal surface of a permanent first molar under local anaesthetic. Dental pain was assessed using the faces pain scale before (dental pain expectation), immediately after (dental pain perception) and six weeks after (memory of dental pain) the dental procedure. Dental pain expectation scores were significantly higher than dental pain perception, independent of the levels of stress, state anxiety and trait anxiety. Children with high scores of stress (OR 1.05 95%CI 1.02–1.09), state anxiety (OR 1.15 95%CI 1.05–1.27) and trait anxiety (OR 1.18 95%CI 1.07–1.30) were more likely to report greater scores of dental pain expectation. Children anticipated more dental pain than what was actually perceived after the dental restoration. Children with greater levels of stress and anxiety have a distorted evaluation of expected dental pain before the dental procedure.

## 1. Introduction

Pain can be defined as a “noticeably uncomfortable and emotional experience associated with actual or potential tissue damage, or described at the time of such damage” [1]. As suggested by this definition, pain is always a subjective event, and the perception of pain is related to physiological, psychological, social and cultural factors [2]. Experiences of pain in childhood depend on the integration of noxious stimuli, behavioural conditions, cognitive, affective and psychological components of the individuals in the context of their personality development and socio-cultural environment [3]. Furthermore, it is acknowledged that variance in pain sensitivity is partially mediated by genetic factors [4].

Psychological factors of pain include emotional and cognitive aspects. They may exacerbate the perception of pain due to catastrophic thinking about pain, anxiety and fear associated with pain. Otherwise, self-esteem, coping strategies, self-efficacy and self-reliance are psychosocial factors that may reduce pain [1]. Pain is strongly linked to anxiety, stress, mood disorders and depression since the prevalence of these psychological disorders are higher among individuals with perceived chronic pain than in the general population [5,6,7,8,9,10,11]. The co-occurrence of pain and psychological problems is higher than when these conditions are assessed individually. In addition, the co-occurrence of pain and psychological problems is frequently associated with poorer quality of life and treatment outcomes, disability, and inadequate health care utilization [5,6,8,11]. The influence of psychological factors on the occurrence of pain is supported by evidence from studies assessing the relationship of anxiety disorders and depressive symptoms with unexplained chronic pain [7], non-specific chronic pain [8,11], musculoskeletal pain [10,12], headaches [9,10] and stomach pain [9]. In contrast, dental fear and dental anxiety are the main psychosocial factors which have been investigated concerning dental pain [13].

Children may be subject to painful experiences as a result of clinical treatment and related procedures for the management of oral and dental conditions. Pain experiences from dental treatment in children can generate different reactions, which may affect their behaviour in the dental office and increase dental anxiety [14,15]. In fact, this illustrates the compound and subjective nature of pain and suggests the importance of psychosocial factors to understand the complexity between dental treatment and dental pain [14,16,17,18,19,20].

Previous studies on the perception of dental pain during dental clinical procedures indicated that adolescents and adults attribute their current level of dental phobia to previous painful experiences in the course of dental treatment during their childhood [18,21,22]. A systematic review also suggested that patients who have experienced pain even only occasionally during dental treatment are more likely to remain afraid of receiving future dental care in spite of many subsequent pain-free dental visits [23]. Unpleasant experiences during dental treatment may result in dental distress (i.e., negative emotions linked to dental stress) [24] that occurs when a person prepares him/herself to cope with future stress in the forthcoming dental visits due to previous undesirable events [25].

Previous studies about the memory of pain in children concluded that children’s behaviour in potentially “difficult” medical situations may be determined by the nature of their memories concerning their former experiences in similar environments, such as in dental settings [22]. The facility or difficulty in coping with these potentially challenging situations varies according to people’s memories, resources and experiences [26].

The present study was conducted to improve the understanding of the role of general psychological disorders on dental pain expectation, pain perception and memory of pain. The justification for conducting this research was based on the following aspects. First, previous evidence on this topic focused mainly on the link between psychological problems and chronic pain. Secondly, research on psychosocial factors and children’s dental pain was predominantly devoted to dental anxiety. The aim of this study was to assess the association of stress and anxiety with the expectation, perception and memory of dental pain in children. Furthermore, this study was carried out to determine whether children anticipate more discomfort than they actually experience and whether they recall more discomfort six weeks later than they report immediately after their dental treatment.

## 2. Material and Methods

### 2.1. Study Design and Participants

This was a six-month follow-up study conducted in a state-funded school in Petropolis. Petropolis is a city with nearly 300,000 inhabitants, in the Rio de Janeiro state, Brazil.

The public school had a dental office where a paediatric dentist (investigator) routinely provided oral health prevention and dental treatment to students. A convenience sample of students attending between year one and year four was selected. Initially, all students aged 9 to 12 years (N = 132) were consecutively assessed for inclusion. The eligibility criteria were children who had a decayed tooth with cavity in the occlusal surface on a permanent upper or lower, right or left first molar, and children who had never received previous dental treatment by the investigator. Children who received dental treatment during the last three months (N = 67), those with spontaneous dental pain (N = 10), and those who were currently using analgesics to treat some illness (N = 6) or under psychological/psychiatric treatment (N = 3) were excluded. Therefore, of the 132 invited children, 46 children met the eligibility criteria. All invited parents agreed to the participation of their children. The mean age of the participants was 10.6 (SD = 1.0) years old, and 50.0% of the sample were female children. Male children were older than females. The majority of children were in the 4th year (Table 1).

### 2.2. Data Collection

Before the main study, six children attending the same school participated in the pilot study to test the understanding of the items of the questionnaire and to test the clinical protocol of the dental procedure. No changes were needed.

One trained paediatric dentist (investigator) selected the potential participants according to the eligibility criteria. A formal invitation letter explaining the aims and procedures involved in the study was sent to the students’ parents. All approved their children’s participation in the study and signed a written informed consent. The present study was conducted in accordance with the Declaration of Helsinki and the research protocol was approved by the Research Ethics Committee of the Federal University of Rio de Janeiro.

Data collection, including the six-week follow-up interview, was conducted by the investigator at the dental office located in the school. Initially, the participants were individually interviewed to obtain demographic (age and sex) data and to complete the questionnaires to assess their levels of stress and anxiety. After that, each child received a detailed explanation of the dental treatment to restore the decayed tooth, including the local aesthetic procedure. Then, they completed the dental pain scale to assess the expectation of dental pain. Immediately after dental treatment, which involved the injection of local anaesthetic, preparation of a cavity on the occlusal surface of a permanent first molar and restoration with amalgam, participants were asked to rate the degree of pain which they had just experienced to assess the perception of dental pain. Six weeks later, they answered the dental pain scale again in order to evaluate the memory of dental pain, recalling the pain they had experienced during the previous treatment. Each child was interviewed and received dental treatment individually in a private dental office at school. There was no distinction between pain during the local anaesthetic and the pain from the dental procedure itself.

### 2.3. Measures

#### 2.3.1. Dental Pain

The faces pain scale is a 7-point scale comprising seven faces that depicts dental pain severity into different graduations, ranging from “no pain” on the leftmost face to “worst pain possible” on the rightmost face. Previous data shows that the face ‘1’ corresponds to “no pain”, and the face ‘7’ corresponds to “the worst pain possible”, similar to a visual analogue scale (VAS) [27]. The scale is simple and easy to apply, requiring minimal instructions. To assess dental pain expectation, the interviewer read the following instruction before the dental procedure: “Please look at these faces. They show how much pain a person can feel. Can you point out the face that shows how much pain you are expecting?” Dental pain perception was evaluated immediately after the dental procedure, when they were asked to point out the face that represented how much pain they have experienced. Dental pain memory was assessed six weeks later when they were inquired to choose the face that reflected how much pain they experienced during the previous dental treatment.

#### 2.3.2. Stress

Stress was measured using the children’s stress scale [28]. This scale was developed and validated for Brazilian children aged 6 to 14 years of both genders to assess the occurrence of clinical condition of stress, according to four dimensions of stress: physical, psychological, psychophysiological and psychological with depressive components. The scale included items such as “My heart beats fast, even when I do not run or jump”; “I have trouble breathing”; and “I have trouble paying attention”. The answers to the items are given by the children using a 5-point Likert scale, which presents the frequency that they experience stress symptoms: never = 1; a little = 2; sometimes = 3; almost always = 4; always = 5. The higher the scores on the scale, the more significant the physical and psychological manifestations of stress. A single total score was computed for each child and the children were divided into two groups: those with less than 52.5 (considered unstressed) and those with 52.5 or more (considered stressed). The above-mentioned cut-off points were based on clinical observations and recommended by the authors of the instrument [28].

#### 2.3.3. State–Trait Anxiety

Anxiety was assessed using the state–trait anxiety inventory for children [29]. The total score of trait and state anxiety scales can vary between 20 and 60. The state anxiety scale consists of 20 items, such as “I feel very nervous/I feel nervous/I do not feel nervous”, “I feel very agitated/I feel agitated/I do not feel agitated”, and “I feel very worried/I feel worried/I do not feel very worried”. The written instructions ask the child to report how he/she feels on each item. This scale measures the short-term and transitory state anxiety, which is specific to a given situation at a particular moment in time. The children were divided into two groups of state anxiety according to the median of the total score. Those with scores varying from 24 to 29 were called low scores and patients with scores from 30 to 53 were considered high scores.

The trait anxiety scale also consists of 20 items, such as “I keep thinking about things that are not important” and “I get worried about things that can happen”, which indicate a general proneness to anxious behaviour rooted in the personality. The written instructions were read to the child asking how he/she feels in general. Then, he/she chooses one of the three alternatives, which vary from “almost never” to “frequently”. A total score was derived from each child’s answers and the children were divided, using the median, into two groups of trait anxiety. Participants with scores from 29 to 38 were considered low scores and those with scores from 39 to 53 were considered high scores.

### 2.4. Statistical Analysis

Dental pain scores and psychosocial measures were not normally distributed according to the Shapiro–Wilk test (*p* < 0.05). The comparison of age, education, psychosocial measures, expectation, perception and memory of dental pain between sex groups was checked using Mann–Whitney and Fisher’s exact tests. Children’s stress scale and state–trait anxiety inventory psychometric properties were assessed for internal consistency using Cronbach α coefficient and 95% confidence intervals (95% CI). Statistical correlations between psychosocial factors and dental pain measures were sought after using the Spearman coefficient correlation. The Mann–Whitney test was used to compare the expectation, perception and memory of dental pain measures between stress, state anxiety and trait anxiety groups. Pairwise comparisons between dental pain expectation, dental pain perception and dental pain memory for the whole sample and relevant psychosocial factor groups were assessed using the Wilcoxon test. Multivariate ordinal regression was carried out to estimate the odds ratio with 95% CI and *p* values of stress, state anxiety, trait anxiety and dental pain expectation, dental pain perception and dental pain memory adjusted for age and sex. All analyses were performed in IBM SPSS Statistics version 24.0 (IBM Corp., Chicago, IL, USA). The significance level established for all analyses was 5% (*p* ≤ 0.05).

### 2.5. Study Power

The study power was estimated as 80% based on the sample size of 46 children considering a significance level of 5% to detect a coefficient correlation of at least 0.40.

## 3. Results

Stress and state anxiety scores were significantly higher in females than males. The mean scores of stress, state anxiety and trait anxiety were 43.8 (SD = 1.0), 31.2 (SD = 7.1) and 39.7 (SD = 6.3), respectively. The means of the faces pain scale before, immediately after and 6 weeks after the dental procedure were 3.9 (SD = 2.5), 1.7 (SD = 1.1) and 2.2 (SD = 1.4), respectively (Table 1).

The Cronbach’s alpha of the instruments employed to assess stress, state anxiety and trait anxiety were 0.863 (95% CI 0.799–0.914), 0.845 (95% CI 0.772–0.903), and 0.782 (95% CI 0.679–0.864), respectively. Stress, state anxiety and trait anxiety were positively correlated with dental pain expectation (*ρ* = 0.459, *p* = 0.001; *ρ* = 0.504, *p* < 0.001; *ρ* = 0.412, *p* = 0.004, respectively). Substantial correlations were also observed between stress, state anxiety and trait anxiety (*p* < 0.01) (Table 2).

Dental pain expectation differed between stress and anxiety groups. Children with stress and high scores of state and trait anxiety had greater dental pain expectation than those from unstressed and low levels of anxiety groups (*p* ≤ 0.05). Overall, participants anticipated more dental pain (3.91 ± 2.47) than what was actually perceived (1.67 ± 1.06), independent from their levels of stress and anxiety (*p* < 0.01). Dental pain expectation was also significantly higher than memory of dental pain 6 weeks later (mean = 2.17 ± 1.40) (*p* < 0.001). This finding was similar across all psychosocial factors subgroups, except in the low state anxiety group. Memory of dental pain was higher than the perception of dental pain among children with high trait anxiety (Table 3).

**Figure 1:**
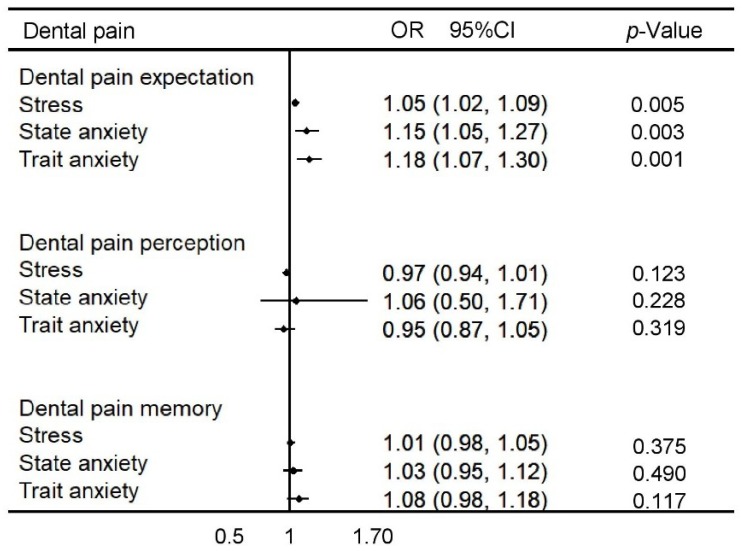
The association of stress, state anxiety and trait anxiety with dental pain measures adjusted by age and sex. (OR: odds ratio; 95% CI: 95% confidence interval).

The association of stress, state anxiety and trait anxiety with dental pain expectation, perception of dental pain and memory adjusted for age and sex is summarised in Figure 1. Children with high levels of stress (OR 1.05 95% CI 1.02–1.09), state anxiety (OR 1.15 95% CI 1.05–1.27) and trait anxiety (OR 1.18 95% CI 1.07–1.30) were more likely to report a higher expectation of dental pain. The investigated psychosocial factors were not significantly associated with dental pain perception and memory of dental pain.

## 4. Discussion

This study examined the role of stress and anxiety on children’s expectation, perception and memory of dental pain related to a restorative procedure in a permanent decayed tooth. The findings from the present study suggest that children with higher levels of stress and anxiety had a greater expectation of dental pain just before a dental restorative procedure. Thus, stress and anxiety were relevant psychological factors associated with dental pain expectation in children. On the other hand, contrary to what was anticipated on the basis of the literature [13,14,30,31], stress and anxiety did not influence children’s perception of dental pain immediately after the dental procedure, nor the memory of dental pain six weeks after dental treatment.

The anticipated dental pain before the dental procedure was greater than the perceived dental pain reported immediately after treatment, independent from the participants’ levels of stress, state anxiety and trait anxiety. The expectation of dental pain was also higher than dental pain-recall 6 weeks after dental treatment in the whole sample and across the stress and anxiety subgroups, except among those with low levels of state anxiety. The findings from the current study support the hypothesis that children anticipate more pain than they experience during restorative dental treatment apart from their stress and anxiety status. The discrepancy between expectation and perception of dental pain has been reported in adult patients [17,19,32]. A previous study in adults showed no significant differences between anticipated and perceived pain, although patients with high anxiety reported less perceived than expected dental pain [32]. The possible explanation for the discrepancy refers to different age groups which in turn influence the accuracy of the reports of anticipated and experienced pain.

Similar to the present findings in children, anxiety was a meaningful factor on the prediction of dental pain in adults since subjects with high anxiety expected more dental pain than those with low anxiety [17,32]. Contrary to our results, perception of dental pain was substantially different between adults with different levels of anxiety [17,19,32]. The unrealistic expectations of dental pain among children may result from uncomfortable or unpleasant previous dental treatment experiences, dental fear or from parents’ dental anxiety [14,16,18]. Other factors may also have contributed to the high expectation of dental pain among the participants of this study. First, a detailed explanation of the dental treatment was provided by the dentist before completing the dental pain scale. Dental pain expectation is higher when patients received restorative treatment or dental extraction than when they receive a check-up [32]. Second, the dental pain scale before dental treatment was completed in the dental office where the dental procedure would be conducted. Thus, children were exposed to the dental environment including the dental equipment and dental instruments when completing the dental pain scale. Finally, children were asked to fill the anxiety and stress questionnaires immediately before the dental procedure, which is an unusual task in dental appointments that may have exacerbated their expectation of dental pain. It should also be acknowledged that some of the aforementioned aspects might have also influenced the stress and anxiety scores. However, the dental environment should not be considered the primary factor that influenced evaluations of dental pain and distorted psychological measures. Maternal dental fear and anxiety, history of toothache and frequency of dental visits were also meaningful predictors of dental pain and dental anxiety in children and adults [14,16,17,19,32].

One of the hypotheses of this study was that the perception of dental pain immediately after the restorative dental procedure would be lower than the memory of dental pain six weeks later. However, the difference between the scores in the whole sample was marginally non-significant. When the aforementioned comparison was tested across the groups with different levels of stress and anxiety, the hypothesis that children recall more dental pain than they actually perceived was only confirmed among children with high trait anxiety. The present study showed a positive correlation between stress, state anxiety, trait anxiety and dental pain expectation. In addition, the anticipated dental pain of the stressed group, high state anxiety and high trait anxiety was significantly higher than the respective control groups. Although these findings have been already reported in adults, evidence on the link between psychosocial factors and dental pain expectation in children is scarce [17,19,20,32]. It can be argued that stress and anxiety can negatively affect children’s emotions and perceptions when facing a potential negative situation related to dental treatment influencing how they appraise the subjective aspects of dental care. The cognitive process related to anticipation of dental pain on stressed and anxious subjects promotes a negative expectation, changing the sensorial quality of pain.

The following methodological limitations of the present study should be acknowledged. The small sample size and convenience sample suggest that the findings should not be generalized. In addition, the sample was selected in a public school in Brazil which is predominantly composed of students from low-socioeconomic families. Therefore, our findings on the association of stress and anxiety with the expectation of dental pain might be overestimated since children’s levels of stress and expectation of dental pain may correlate with low family socioeconomic status. The participants were divided into low and high state and trait anxiety groups according to the median of the anxiety scores. Although this was needed for analytical purposes, such an approach imposes restrictions on the generalization of the results. Our findings might be influenced by information bias with regards to dental pain measurements, since a single dentist collected the baseline data, provided the dental treatment and conducted the six-week follow-up interview. Furthermore, information concerning previous dental treatments which might have influenced the expectation of dental pain were not collected. The findings should be interpreted with caution since the dental intervention was a restorative procedure in a permanent decayed tooth. Possibly, the results would differ if the dental intervention would be a dental check-up or a more invasive dental procedure (e.g., tooth extraction).

Future research on the influence of psychosocial factors on dental pain should consider depressive symptoms, since depression is a relevant psychological problem among children [7]. In addition, involving children from different socioeconomic background would generate more generalizable results. Although children who received dental treatment during the last three months were excluded, information concerning previous dental treatments could be collected from the parents and included in the analysis. Parents’ perception of children’s dental pain and their psychological characteristics would enhance the understanding of the complex interrelationship between psychological factors and dental pain in children. Dental restorative treatment in permanent teeth is one of the most common dental procedures in children; however, other types of dental treatments as well as dental interventions in primary teeth could be considered in future research.

## 5. Conclusions

Children anticipated more dental pain than they perceived after a restorative dental procedure. Furthermore, high levels of stress and anxiety are capable of inducing a distorted evaluation of the degree of expected dental pain in children, which can predispose children to disruptive behaviour during dental appointments.

## Figures and Tables

**Table 1:** Demographic characteristics, stress and anxiety of participants according to sex.

Variable	Total	Males	Females	*p*-Value
Age, mean (SD)	10.6 (1.0)	10.9 (0.9)	10.2 (1.0)	0.019 ^a^
Education, N (%)				0.091 ^b^
1st	10 (21.7)	8 (34.8)	2 (8.7)	
2nd	13 (28.3)	6 (26.1)	7 (30.4)	
3rd	9 (19.6)	2 (8.7)	7 (30.4)	
4th	14 (30.4)	7 (30.4)	7 (30.4)	
Stress				
Mean (SD)	43.8 (18.9)	36.7 (14.6)	50.9 (20.3)	0.014 ^a^
Non-stressed, N (%)	30 (65.2)	19 (82.6)	11 (47.8)	0.029 ^b^
Stressed, N (%)	16 (34.8)	4 (17.4)	12 (52.2)	
State anxiety				
Mean (SD)	31.2 (7.1)	28.6 (3.6)	33.8 (8.7)	0.048 ^a^
Low scores	23 (50)	13 (56.5)	10 (43.5)	0.278 ^b^
High scores	23 (50)	10 (43.5)	13 (56.5)	
Trait anxiety				
Mean (SD)	39.7 (6.3)	38.0 (5.8)	41.4 (6.5)	0.101 ^a^
Low scores	23 (50)	14 (60.9)	9 (39.1)	0.238 ^b^
High scores	23 (50)	9 (39.1)	14 (60.9)	
Faces pain scale, mean (SD)				
Expectation	3.9 (2.5)	3.5 (2.5)	4.3 (2.5)	0.234 ^a^
Perception	1.7 (1.1)	1.5 (0.7)	1.8 (1.3)	0.439 ^a^
Memory	2.2 (1.4)	2.3 (1.4)	2.1 (1.4)	0.497 ^a^

^a^: *p*-Value refers to Mann–Whitney test for comparison between gender groups. ^b^: *p*-Value refers to Fisher Exact test for comparison between gender groups.

**Table 2:** Correlation matrix (Spearman coefficient) between stress, state anxiety and trait anxiety and dental pain measures.

Variables	Dental Pain Expectation	Dental Pain Perception	Dental Pain Memory	Stress	State Anxiety	Trait Anxiety
**Dental Pain Expectation**	1					
**Dental Pain Perception**	0.035 (*p* = 0.818)	1				
**Dental Pain Memory**	0.200 (*p* = 0.182)	0.091 (*p* = 0.550)	1			
**Stress**	0.459 (*p* = 0.001) *	−0.133 (*p* = 0.379)	0.101 (*p* = 0.502)	1		
**State Anxiety**	0.504 (*p* < 0.001) *	0.268 (*p* = 0.071)	0.226 (*p* = 0.131)	0.403 (*p* = 0.005) *	1	
**Trait Anxiety**	0.412 (*p* = 0.004) *	−0.124 (*p* = 0.410)	0.184 (*p* = 0.220)	0.701 (*p* < 0.001) *	0.399 (*p* = 0.006) *	1

* *p*-Value ≤ 0.05.

**Table 3:** Dental pain expectation, dental pain perception and dental pain memory according to stress, trait and state anxiety groups.

Variables	Total	Stress		State Anxiety		Trait Anxiety	
		Non-stressed	Stressed	Low score	High score	Low score	High score
Dental Pain Expectation ^a^	3.91 ± 2.48	3.33 ± 2.32	5.00 ± 2.45	2.87 ± 2.20	4.96 ± 2.33	3.22 ± 2.37	4.61 ± 2.43
		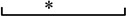		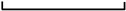		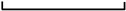	
Dental Pain Perception ^b^	1.67 ± 1.06	1.80 ± 1.24	1.44 ± 0.51	1.96 ± 1.36	1.39 ± 0.50	1.91 ± 1.35	1.43 ± 0.59
Dental Pain Memory ^c^	2.17 ± 1.40	2.20 ± 1.40	2.13 ± 1.45	2.43 ± 1.47	1.91 ± 1.31	1.91 ± 1.16	2.43 ± 1.59
*p*-Value a × b **	<0.001	0.002	0.002	0.008	<0.001	0.009	<0.001
*p*-Value a × c **	<0.001	0.008	0.004	0.062	0.001	0.004	0.003
*p*-Value b × c **	0.057	0.266	0.088	0.059	0.336	0.684	0.006

* *p*-Value ≤ 0.05 refers to Mann-Whitney test for comparison within groups. ** *p*-Value refers to Wilcoxon test for comparison between dental pain groups.
